# How people think about the truth of hypothetical impossibilities

**DOI:** 10.3758/s13421-023-01454-y

**Published:** 2023-10-03

**Authors:** Ruth M. J. Byrne

**Affiliations:** School of Psychology and Institute of Neuroscience, Trinity College Dublin, University of Dublin, Dublin, Ireland

**Keywords:** Impossibility, Counterfactual, Conditionals, Simulation

## Abstract

People can think about hypothetical impossibilities and a curious observation is that some impossible conditionals seem true and others do not. Four experiments test the proposal that people think about impossibilities just as they do possibilities, by attempting to construct a consistent simulation of the impossible conjecture with its suggested outcome, informed by their knowledge of the real world. The results show that participants judge some impossible conditionals true with one outcome, for example, “*if people were made of steel, they would not bruise easily*” and false with the opposite outcome, *“if people were made of steel they would bruise easily*”, and others false with either outcome, for example, “*if houses were made of spaghetti, their engines would (not) be noisy*”. However, they can sometimes judge impossible conditionals true with either outcome, for example, “*if Plato were identical to Socrates, he would (not) have a small nose*”, or *“if sheep and wolves were alike, they would (not) eat grass”*. The results were observed for judgments about what *could be* true (Experiments 1 and 4), judgments of degrees of truth (Experiment 2), and judgments of what *is* true (Experiment 3). The results rule out the idea that people evaluate the truth of a hypothetical impossibility by relying on cognitive processes that compare the probability of each conditional to its counterpart with the opposite outcome.

## Introduction

Some impossibilities seem true, such as “*if people were made of steel, they would not bruise easily*”, whereas others seem false, such as “*if people were made of steel, they would bruise easily*”. Granted that both conjectures are impossible (people cannot be made of steel), why does one conditional seem true and the other, not? I aimed to examine how people assess the truth of a conditional about an impossibility, to test the proposal that they simulate it in the same way as they simulate a conditional about a possibility, guided by their knowledge of the real world. Hundreds of empirical studies over many decades have examined how people understand conditionals about facts, such as “*if the lake was cold, they did not swim in it*” (see Nickerson, 2015, for a review). Likewise, hundreds of empirical studies more recently have examined how people understand counterfactual conditionals, about matters that once were possible but are so no longer, such as “*if the lake had been polluted, they would not have swum in it*” (see Byrne, 2016, for a review). Perhaps surprisingly, very little attention has been given to how people understand conditionals about impossibilities, such as “*if lakes were made of bleach, people would not swim in them*”. Yet arguably a full account of how people engage in hypothetical thinking requires an understanding of how they think not only about facts and possibilities (e.g., Byrne & Johnson-Laird, 2020; Khemlani et al., 2018), but also about impossibilities (e.g., De Brigard & Parikh, 2019; Phillips et al., 2019; Phillips & Cushman, 2017). There is as yet no consensus about the cognitive processes that underlie conditional reasoning, and whether people think hypothetically by constructing small scale models of a situation and searching for counterexamples to putative conclusions (e.g., Johnson-Laird et al., 2015; Khemlani et al., 2013, 2018), or whether they rely instead on their beliefs to calculate the conditional probability of a situation (e.g., Evans & Over, 2004; Oaksford & Chater, 2007; Over et al., 2007). Moreover, there are currently no psychological theories about how people reason with conditionals about impossibilities. Of course, there are many different sorts of impossibilities, from logical, to epistemical, to metaphysical impossibilities. And although adults, and children, can make inferences from premises presented as fantastical fiction, for example, about an alien planet, or about a make-believe pretend animal (e.g., Amsel et al., 2005; Dias & Harris, 1988; Markovits et al., 2010), little is known about how they interpret the meaning of such premises, or whether they are willing to judge them to be true or false.

 My starting proposal is that people reason with conditionals about impossibilities by drawing on their knowledge about the real world to attempt to construct a consistent simulation of an impossible conjecture and its suggested outcome. The conditional, “*if people were made of steel, they would not bruise easily*” seems true because a salient property of steel is that it is hard, and hard substances are known not to bruise, so reasoners can readily construct a simulation of steel-people who do not bruise. Its counterpart with the opposite outcome, steel-people who bruise easily, seems false because reasoners cannot readily construct a consistent simulation given their real-world knowledge. Consider the related example, “*if people were made of peaches, they would not bruise easily*”. It seems false, because a salient property of peaches is that they are soft, known to bruise, and so reasoners cannot readily construct a simulation of peach-people who do not bruise, given their real-world knowledge. Only one consistent simulation is possible in each case for the steel-people and peach-people examples, and so only one of the two conditionals seems true for this sort of *known-reality* content (we know that in reality people bruise, and we know about the relevant properties of steel, or of peaches, in this context).

 My proposal about how people understand impossible conditionals derives from the theory that people understand conditionals generally by simulating a small-scale model of how the world would be if an assertion were true (e.g., Byrne & Johnson-Laird, 2020; Johnson-Laird & Byrne, 2002). For a conditional about matters of fact, such as *“If there was an apple in the fruit bowl, there was a banana”,* people initially create an intuitive simulation of the possibility in which there was an apple and a banana, and they can also deliberate further about other possibilities consistent with the conditional, for example, it is also possible that there was no apple and no banana (e.g., Khemlani et al., 2018). Their knowledge modulates their models, eliminating possibilities known to be untrue, or adding ones known to be true, for example, some reasoners may consider that it is also possible there was no apple, but there was a banana. Hence the cognitive processes that assess the truth of a conditional rely on thinking about several possibilities, for example, it is possible that there was an apple and a banana, and it is possible that there was no apple and no banana. If further information is acquired, for example, that there was indeed an apple in the fruit-bowl, the epistemic status of the first possibility can be updated to indicate that it corresponds to the facts, and the second possibility can be updated to indicate that it was once possible but is no longer so.

For a counterfactual conditional, about matters that once were possible but are so no longer, for example, *“If there had been an apple in the fruit bowl, there would have been a banana”,* people construct a model of the possibility in which there was an apple and a banana, but they also recover the presupposed or known facts, there was no apple and no banana. They keep track of the epistemic status of these possibilities, that the first corresponds to the counterfactual conjecture, once possible but no longer so, and the second corresponds to the facts (e.g., Byrne, 2005). Because they have mentally represented more possibilities from the outset to understand the counterfactual than the factual conditional, people make different inferences from them, their latencies to comprehend their consequences differ, and people even look at different images when they hear them (e.g., Ferguson & Sanford, 2008; Ferguson et al., 2008; Orenes et al., 2022; Santamaria et al., 2005; Thompson & Byrne, 2002). And just as knowledge modulates their models of factual conditionals, so too it can add or eliminate models for their mental representation of counterfactuals (e.g., Espino & Byrne, 2021).

Conditionals about impossibilities can seem very different to counterfactuals about matters that once were possible but are so no longer. The counterfactual’s meaning can be assessed by creating an alternative to reality, just like reality in every way, except that, for example, there is an apple in the fruit bowl. In contrast, logicians have argued that the meaning of a *counterpossible*, a conditional with an impossible conjecture, cannot be assessed in a logic based on constructing a “possible world” (e.g., Lewis, 1973; Stalnaker, 1968; see Williamson, 2018, 2020). Moreover, in everyday life, ordinary reasoners rarely construct “miracle world” conditionals, such as *“if there hadn’t been gravity, the ‘plane wouldn’t have crashed”* compared to the frequency with which they construct “close” counterfactuals, such as, *“if there hadn’t been a bird strike, the ‘plane wouldn’t have crashed”* (e.g., De Brigard et al., 2013; Dixon & Byrne, 2011; Kahneman & Tversky, 1982; Markman et al., 2008; Roese & Epstude, 2017). Notwithstanding such differences, my first step in examining how people think about impossible conditionals is to propose that people do indeed assess the meaning of an impossible conditional just as they do the meaning of a counterfactual, by simulating an alternative that is just like reality in every way except, say, people were made of steel.

Is it correct to consider a conditional about an impossibility true, or false? Prominent philosophical analyses indicate that the normative standard should be that conditionals with impossible conjectures are all vacuously true, and they can lead logically to any outcome (e.g., Williamson, 2018, 2020). Consider the counterfactual, *“If Bizet and Verdi had been of the same nationality, they both would have been French”* (Quine, 1950). There seems to be no way to argue it is true, and the opposite, *“If Bizet and Verdi had been of the same nationality, they both would have been Italian”* is false. The tactic of temporarily supposing that the antecedent is true and evaluating the consequent in that context (Ramsey, 1929; see also Stalnaker, 1968; Lewis, 1973), works equally well for both counterfactuals. Hence the impossible conjecture must be true with either consequent. The conclusion that *all* counterfactuals with impossible conjectures are vacuously true continues to hold considerable sway philosophically (e.g., Williamson, 2018, 2020). And yet for counterfactuals about matters that once were possible but are so no longer, people readily distinguish between ones that are true, for example, *“if he had had two aces, he would have won”*, and ones that are false, *“if he had had two 2’s, he would have won”* (Byrne & Johnson-Laird, 2020). They can construct a consistent simulation of the possibilities corresponding to the conjecture and the presupposed facts for the first counterfactual, whereas for the second, their knowledge of the card game rules out the possibility corresponding to the conjecture. Hence, if people think about counterfactuals about impossible conjectures in a similar way, we can expect they will distinguish some counterpossibles that are true and some that are false.

However, that people do not appear to interpret impossible conditionals as vacuously true has been ascribed to a cognitive bias (Williamson, 2018, 2020). The suggestion of a cognitive bias underlying how people understand impossible conjectures has been traced to the idea people understand conditionals by relying on a conditional probability comparison (Williamson, 2018). On the conditional probability account, people assess a conditional about matters of fact, for example, “*If there was an apple, there was a banana*” not by constructing models of possibilities, but by comparing the strength of their prior belief that there was a banana, given there was an apple, *P (B|A),* to their prior belief that there was no banana, given there was an apple*, P (not-B|A).* The meaning of a conditional is given by a “Ramsey test” of supposing the antecedent is true and evaluating the consequent in this context (Ramsey, 1929; see Evans & Over, 2004; Oaksford & Chater, 2007). Its meaning corresponds to a "defective" truth table, in which the conditional is true when its antecedent and consequent are true, false when its antecedent is true and its consequent false, and the conditional has no truth value in the other two instances in which the antecedent is false (e.g., Evans & Over, 2004).

On this probabilist view, the cognitive processes that assess the truth of a factual conditional rely on a comparison of the situation in which, for example, there was an apple and a banana, to the situation in which there was an apple and no banana, weighing up a person’s relative beliefs in the probability of each one. Similarly, the assessment of a counterfactual depends on a person’s prior beliefs (e.g., Over et al., 2007; see also Lucas & Kemp, 2015; Lagnado et al., 2013; Meder et al., 2010). Accordingly, it has been proposed that people are led astray by the conditional probability comparison process to believe that a conditional about an impossibility seems true while its counterpart with the opposite outcome seems false (Williamson, 2018, 2020). For example, when they apply the comparison to an impossible conditional, such as the well-known case, *“if Hobbes had squared the circle, sick children in South America at the time would not have cared”* (Nolan, 1997), they mistakenly conclude that since it seems true, its counterpart must be false, *“if Hobbes had squared the circle, sick children in South America at the time would have cared”* (Williamson, 2018). On this account it is a mistake to consider an impossible conditional true with one outcome and false with the other, and the mistake arises because a conditional probability judgment implies that people assess the truth of a conditional by making a comparison of their beliefs about one conditional, such as the one about an apple and a banana, to its counterpart with the opposite outcome, about an apple and no banana.

This application of a conditional probability comparison to impossible conditionals implies that people will tend to judge an impossible conditional true with only one outcome, and false with the opposite outcome (Williamson, 2018, 2020). But what if people do not rely on such a comparison process when they understand impossible conditionals? My starting position that people simulate small-scale models of possibilities does not imply they assess truth by comparing the impossible conjecture with one outcome to the impossible conjecture with the opposite outcome. In fact, according to the model theory, when people understand a conditional, they tend *not* to think about its antecedent with the opposite outcome. For example, people think about the possibilities consistent with a factual conditional, such as an apple and a banana, no apple and no banana, even no apple and a banana, but they do *not* think about the possibility of an apple and no banana (Johnson-Laird & Byrne, 2002; Khemlani et al., 2018). They assess the truth of the conditional about an apple and a banana without ever thinking about an apple and no banana. Hence, the application of the model theory to impossible conditionals implies that people are not constrained to judge an impossible conditional true with one outcome only and false with the opposite outcome. Since people can think about whether a conditional is true without having to compare it to its counterpart with the opposite outcome, they should be able to judge that in some cases, a conditional about an impossibility seems true, and its counterpart with the opposite outcome also seems true.

To illustrate, consider another example, “*if Plato were identical to Timaeus, he would not have a small nose*”. It seems plausible that it could be true. But consider the conditional with the opposite outcome, “*if Plato were identical to Timaeus, he* would *have a small nose*”. It too seems as if it could be true. Why does the example about Plato’s nose seem true with both outcomes whereas the steel-people one seems true with only one outcome? For the Plato’s nose example, knowledge of the real world does not eliminate either outcome as a possibility, because we do not know the sizes of Plato’s or Timaeus’ noses. Reasoners can readily construct a simulation of a Timaeus-like Plato with a big nose, and equally, a simulation of a Timaeus-like Plato with a small nose, since the conditionals concern unknowns (the sizes of their noses). Both outcomes seem true because reasoners can construct a consistent simulation of either one of them for such *unknown-reality* content (we do not have sufficient information about the facts to rule out either outcome). A thorny philosophical issue is whether all impossible conditionals should be considered true with both outcomes, i.e., they are logically vacuous (e.g., Williamson, 2018, 2020), or whether some are true with only one, and thus are valid in, say, scientific theorizing (e.g., Berto et al., 2018, 2022; Brogaard & Salerno, 2013; Wilson, 2018, 2021). Psychological evidence about whether people judge some impossible conditionals to be true with both outcomes can contribute to this current philosophical debate. If empirical evidence corroborates the prediction that people can assess that some impossible conjectures, such as those about *unknown-reality* content (e.g., Plato’s nose) could be true for both outcomes, it would go some way to ruling out the idea that a cognitive bias underlies people’s judgments that impossible conjectures with *known* content (e.g., steel-people) are true with only one outcome. It would suggest that their judgments that impossible conditionals are true for only one outcome in such cases do not arise because they rely on a heuristic that prevents them from recognising that impossible conditionals can be true with both outcomes (cf. Williamson, 2018).

Now, consider a third case, “*If houses were made of spaghetti, their engines would not be noisy*”. It seems false, and so too does the opposite, “*If houses were made of spaghetti, their engines* would *be noisy*”. Why do both outcomes seem true for the Plato’s nose example, but neither seems true for the spaghetti-houses one? The question concerns how knowledge of what is possible in the real world constrains the imagination of impossibility. For the spaghetti-houses example, knowledge does not facilitate the construction of a consistent simulation of either outcome (spaghetti houses with noisy engines or not), because the outcomes are unrelated to the impossible conjecture (houses do not have engines). Since reasoners cannot construct a consistent simulation of either of them, they both seem false for such *unrelated-in-reality* content (houses being made of spaghetti and the noisiness of engines are unrelated in the real world). How people distinguish between impossibilities related to reality and those unrelated in reality may shed light on how they keep track of reality (e.g., Espino & Byrne, 2021; Simons et al., 2017), and even on how delusional beliefs are formed (e.g., Coltheart et al., 2011; Ross et al., 2016).

**Fig. 1:**
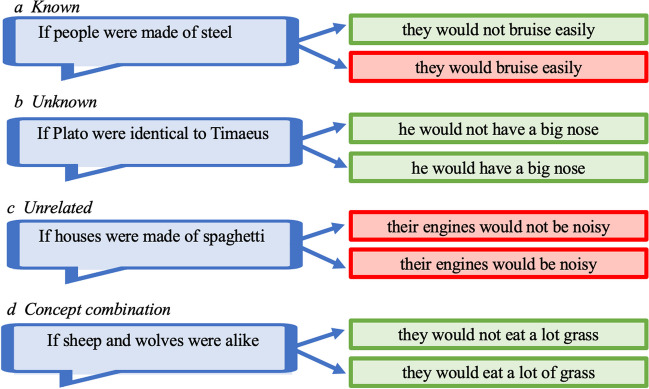
Examples of four categories of content used in the experiments, with an impossible conjecture (in blue), and a suggested outcome and its opposite (those that seem true are in green, and those that seem false are in red). The material set contained four different contents in each category (see Table 1)

 I test three hypotheses in the first three experiments (see Fig. 1):

1. For impossibilities about *known-reality* content, participants will judge a conditional true when they can construct a simulation of its outcome consistent with a salient property of its conjecture (e.g., the bruisability of a substance), and false when they cannot, that is, for its counterpart with the opposite outcome.
2. For impossibilities about *unknown-reality* content, participants will judge a conditional could be true with either outcome given that they can construct a simulation of each one consistent with the conjecture, since the outcome is about a property unknown in reality (e.g., the size of Plato’s nose).
3. For impossibilities about *unrelated-in-reality* content, participants will judge a conditional false with either outcome since both outcomes are unrelated to the conjecture in the real world.

 I test one additional content in the fourth experiment. Consider the example about Bizet and Verdi earlier, *“If Bizet and Verdi had been of the same nationality, they both would have been French”* and, *“If Bizet and Verdi had been of the same nationality, they both would have been Italian”.* Reasoners can construct a consistent simulation anchored on Bizet, in which Verdi is modified to be like him, in which case they both would have been French and the first conditional is true. Equally, they can construct a consistent simulation anchored on Verdi, in which Bizet is modified to be like him, in which case they both would have been Italian and the second conditional is true. Such conditionals are somewhat ambiguous about what the lead topic of the antecedent is, Bizet or Verdi. Novel concept combinations are generally resolved by relying on constraints such as plausibility and informativeness to decide which of two concepts is the lead anchor, and which one is to be modified (e.g., Costello & Keane, 2000). For example, the conditional, “*if sheep and wolves were alike, they would eat a lot of grass*” can be interpreted to be about sheep-like wolves, and reasoners can construct a consistent simulation in which such wolves, modified to be like sheep, eat a lot of grass, and so the conditional is true. But similarly, “*if sheep and wolves were alike, they would not eat a lot of grass*” can be interpreted to be about wolf-like sheep, and reasoners can construct a consistent simulation in which such sheep, modified to be like wolves, do not eat a lot of grass, and so this conditional too is true. The final prediction then is:

4. For impossibilities with flexible *concept combination* content, participants will judge a conditional could be true when they construct a simulation of its outcome consistent with one member of the combined concept in its conjecture; they will judge a conditional could be true with either outcome when they do so for either member of the combined concept.

I test these hypotheses for judgments that an impossible conditional *could be* true or *could be* false in Experiment 1 (and Experiment 4); for judgments of an impossible conditional’s degrees of truth in Experiment 2, and for definite judgments that an impossible conditional *is* true or false in Experiment 3. For impossibilities about *known-reality* content such as the steel-people example*,* and *unrelated-in-reality* content such as the spaghetti-houses example, we expect that people will attempt to construct a consistent simulation modulated by their real-world knowledge, and so their judgments about what *is* true and what *could be* true will be similar. But for impossibilities about *unknown-reality* content such as the Plato’s nose example, their simulations cannot be guided by knowledge of reality (they do not know the size of Plato’s nose), and so their judgments of what *is* true may be more conservative than their judgments of what *could be* true.

## Experiment 1

The aim of the experiment was to examine whether people consider that an impossible conditional could be true when they can construct a simulation of its outcome consistent with its impossible conjecture, and otherwise they consider that it could be false. Since there is as yet no empirical evidence on whether people are willing to consider an impossible conditional as being true or false, the first step relied on modal judgments about whether a conditional about an impossibility *could be* true. If participants attempt to use their real-world knowledge to construct a consistent simulation of an impossible conjecture and its suggested outcome, then they will judge that a conditional could be true with one outcome only, given *known* content, and it could be false with either outcome, given *unrelated* content; they will judge that it could be true with either outcome, given *unknown* content.

### Method

A preregistration of the design and hypotheses for the set of studies of which these four experiments were part is available at the Open Science Framework at https://osf.io/zsntx, using the task described at https://osf.io/kzwce and with materials based on those described there and also at https://osf.io/y87mq.

#### Participants

For each of the experiments, an a priori power test using G*power determined that a sample size of 43 participants would provide 95% power to detect a medium sized effect at p <.05 (and a sample size of 36 participants would provide 90% power). Participants were recruited via Prolific for each experiment and the recruitment stopping rule was set to recruit approximately 10% more participants than required by the power test, in case of attention check failures. Participant recruitment filters were set in each experiment to recruit participants over 18 years of age, with English as their first language, resident in countries with English as the primary language including Australia, Canada, UK, US, Ireland and New Zealand, and for participants not to have taken part in previous studies carried out by the research team. Participants in each experiment were compensated £1.50 sterling for their participation.

The participants in Experiment 1 were 49 volunteers, including 32 women, 16 men, and one person who did not provide gender information, and they had an average age of 34 years, with a range from 18 to 66 years, and one participant did not provide age information. It was intended to eliminate participants if they failed more than two of the four attention checks, or if they incorrectly identified in the memory task an item that had not been presented; no eliminations were required on these criteria in this experiment.

#### Materials and design

The design was fully within-participants. Participants made judgments about conditionals that contained impossible conjectures in three conditions, (a) *known-reality*, (b) *unknown-reality,* and (c) *unrelated-in-reality* content*.* Each participant received four contents in each of the three conditions, that is, 12 contents (see Table 1). Each conditional was presented with two outcomes, which differed only in the presence of “not”, and so each participant received 24 conditionals in total. The content was adapted from philosophical analyses of counterpossible conditionals (e.g., Berto et al., 2018; Williamson, 2018). All the conjectures were intended to refer to metaphysical impossibilities, rather than logical, mathematical, or epistemic ones (Berto et al., 2018, 2022; Brogaard & Salerno, 2013; Williamson, 2018), although whether some are epistemic rather than metaphysical is debatable. The conditionals were in the subjunctive mood, and the present tense.

**Table 1:** Materials used in the experiments

	Impossible conjecture	Outcomes	
*Known-reality*	If people were made of steel	they would not bruise easily	they would bruise easily
	If lakes were made of bleach	people would not swim in them	people would swim in them
	If my goldfish were identical to a shark	he would not bite people	he would bite people
	If her puppy were exactly like a tiger	she would not be afraid of him	she would be afraid of him
*Unknown-reality*	If Plato were identical to Timaeus	Plato would have not a small nose	Plato would have a small nose
	If Aristotle were identical to Heraclitus	Aristotle would not be tall	Aristotle would be tall
	If Socrates were like Cebes	Socrates would not be short	Socrates would be short
	If Sisyphus were like Parmenides	Sisyphus would not have blue eyes	Sisyphus would have blue eyes
*Unrelated-in-reality*	If houses were made of spaghetti	their engines would not be noisy	their engines would be noisy
	If mice were exactly like birds	their horns would not be white	their horns would be white
	If trees were just like grass	their noses would not be made of chickpeas	their noses would be made of chickpeas
	If Mozart were Shakespeare	his horses would not be yellow	his horses would be yellow
*Concept combination*	If sheep and wolves were alike	they would not eat a lot of grass	they would eat a lot of grass
	If lobsters were the same as birds	they would not make cosy nests	they would make cosy nests
	If there were cats on the moon	they would not need spacesuits for oxygen	they would need spacesuits for oxygen
	If elephants were to live under the sea	they would not need gills as well as their long trunks	they would need gills as well as their long trunks

Participants made judgments about an impossible conjecture with just one of its outcomes at a time, and the opposite outcome was presented separately on a different trial, hence there were 24 trials. Trials were presented in a different randomised order to each participant. The participants’ task was to judge the truth of a single conditional and they were told:

For the sentence below please decide whether it could be true or could be false. Please read the sentence carefully.

If people were made of steel, they would bruise easily.

At the outset they were given the general instructions:

“You will be asked to read some sentences that make conjectures such as “if cars were made of rice...” and to make inferences about them. In conversation people often say things that can’t literally be the case, such as “if I were you…” but even so what they say can ring true, or else it can seem to be not true at all. We are interested in how you think about whether such sentences make sense and the sorts of inferences you make about them.”

Four attention checks were interspersed at random with the experimental items which consisted of the sentences: “If you are paying attention, please tick the true box”, and “If you are reading this sentence carefully, please tick the false box”. On the final screen participants were asked to tick only those situations that were mentioned in the study, from a set of five items, four correct and one incorrect.

#### Procedure

Participants were recruited through Prolific and the materials were presented in Alchemer. Participants provided their consent after reading an information sheet, and the experiments received prior approval from the Trinity College Dublin School of Psychology Ethics Committee, Approval ID SPREC092018-9. Each experiment took about 10 min.

#### Information availability

The raw data for the experiments are accessible at the Open Science Framework at https://osf.io/rtcxj/. The study materials are provided in Table 1. The study analysis which was carried out in SPSS is described in full at the start of the results section.

### Results and discussion

The statistical analyses in each experiment were carried out in SPSS and consisted of analysis of variance (ANOVA). Greenhouse-Geiser corrected degrees of freedom were used when the assumption of sphericity was violated. Paired-samples t-tests were used for the pairwise comparisons, with a Bonferroni corrected alpha of p < .0167 for three comparisons in each case.

**Fig. 2:**
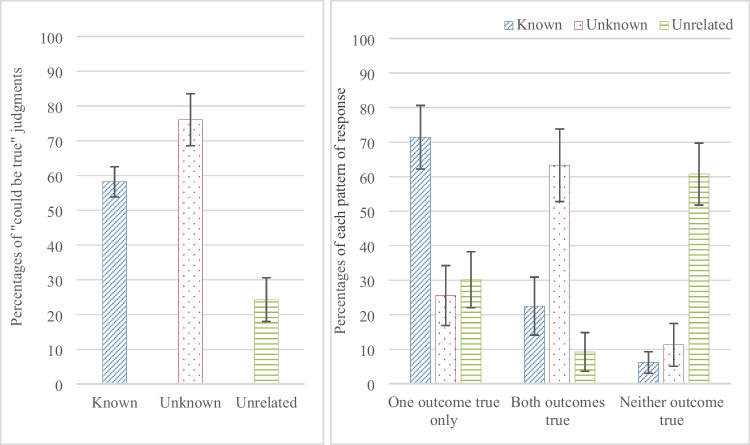
(**a**) The percentages of judgments that an impossible conditional could be true for the three contents of Experiment 1 in which participants made judgments that an impossible conditional *could be* true or *could be* false, and (**b**) the percentages of responses scored for each pair of conditionals that they could be true with one outcome only, with both outcomes, or with neither outcome, for the three contents. Error bars are 95% confidence intervals

We first analyzed judgments that an impossible conditional could be true in a one-way repeated-measures analysis of variance (ANOVA) with the three categories of content (known, unknown, unrelated), on the number of judgments that a conditional could be true. The analysis confirmed that participants made different judgments for the three contents, F (1.673, 80.317) = 79.714, p. < .0001, η_p_^2^=.624; see Fig. 2a. They judged conditionals could be true more often for the *unknown* content compared to the *known* content, t (48) = 4.297, p < .0001, CI [-2.09707, -0.76007], and the *unrelated* content, t (48) = 10.56, p <.0001, CI [3.35405, 4.93166]; and more often for the *known* content than the *unrelated* content, t (48) = 10.342, p < .0001, CI [2.1866, 3.24197].

To explore the patterns of judgments further, we then scored participants’ responses for each of the 12 conditional pairs as indicating that, (a) the two conditionals could be true with one outcome only, (b) the two conditionals could be true with either outcome, or (c) the two conditionals could be true with neither outcome. The one-way repeated-measures ANOVA on their responses that only one outcome could be true showed a significant difference between the three categories of content, F (2, 96) = 44.968, p < .0001, η_p_^2^ = .484, see Fig. 2b. Participants judged that only one outcome could be true more often for the *known* content compared to the *unknown* content, t (48) = 7.792, p < .0001, CI [1.36277, 2.3107], and the *unrelated* content, t (48) = 7.675, p < .0001, CI [1.22002, 2.0861]; there was no difference between the *unknown* and *unrelated* content, t (48) = 0.988, p = .328.^1^

The results show that participants are willing to assess the truth of an impossible conditional in terms of whether it could be true or could be false. They judged that an impossible conditional could be true when they were able to construct a simulation of its outcome consistent with its impossible conjecture, for example, steel people who do not bruise, and false otherwise, for example, steel people who bruise. But when they could construct a consistent simulation of the impossible conjecture with either outcome, they were able to judge that the impossible conditional could be true, and also that its counterpart with the opposite outcome could also be true, for example, Plato-Timaeus with a big nose, or Plato-Timaeus with a small nose. When they could not construct a consistent simulation of the impossible conjecture with either outcome, they judged each impossible conditional to be false, for example, spaghetti houses with noisy engines, or with engines that are not noisy.

Before considering the implications of the results, the next experiment tests whether participants are willing to assess the degrees of truth of an impossible conditional and whether the same results are observed when they do so.

## Experiment 2

The experiment examined participants’ judgments about whether an impossible conjecture *is* true or false, for degrees of truth on a scale that incremented in units of 20 from “False” at 0, to “True” at 100. It tests the hypothesis that participants will judge that a conditional is true with one outcome only, given *known* content; that it is false with either outcome, given unrelated content; and that it is true with either outcome, given *unknown* content.

### Method

#### Participants

The participants were a new set of 46 volunteers recruited from Prolific, who had not taken part in the previous experiment, including 34 women and 12 men, and they had an average age of 33 years, with a range from 18 to 54 years. A further three participants were eliminated, one for failing half of the attention checks and two for providing answers of 0 for 20 of the 24 questions and 100 for the other four questions.

#### Materials, design and procedure

The materials were the same as the previous experiment, but the task was different. Participants were required to make a definite judgment that the conditional is true or false but they provided their judgments on a slider scale labelled “False” at 0, and “True” at 100. The slider button appeared on each screen at 0, it had to be clicked to choose “False” at 0, and moved to choose “True” at 100, or to choose any intervening points, which incremented in units of 20 (illustrated here for a participant’s choice of 40):

For the sentence below please decide whether it is true or false. Please read the sentence carefully.

If people were made of steel, they would not bruise easily

Participants could in effect select one of 6 points, 0, 20, 40, 60, 80, or 100, and this coarse scale was chosen since a finer-grained scale can sometimes lead to inconsistencies in reasoning judgments (Khemlani et al., 2012). Participants carried out three practice trials before the test trials to familiarise them with the slider at the outset, which consisted of instructions to “move the slider scale to true”, “move the slider scale to false” and “move the slider to any number between 0 and 100”. The attention checks were also modified to be, “If you are paying attention please move the slider to 40”, and “If you reading this sentence carefully, please move the slider to 60”. The design and procedure of the experiment were the same as the previous one.

### Results and discussion

**Fig. 3:**
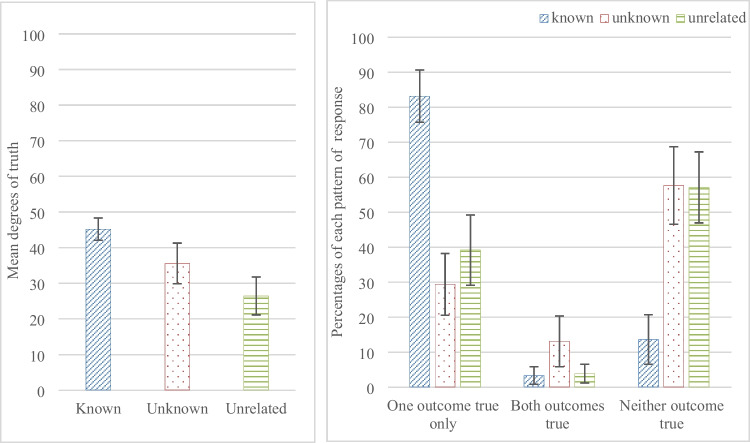
(**a**) The mean degrees of truth participants assigned to an impossible conditional for the three contents of Experiment 2 in which participants assigned degrees on a slider scale, and (**b**) the percentages of responses scored for each pair of conditionals that they are true with one outcome only (0, 20, 40 for one, and 60, 80, 100 for the other), with both outcomes (60, 80, or 100 for both), or with neither outcome (0, 20, or 40 for both), for the three contents. Error bars are 95% confidence intervals

The modal response for known content was 100 for one conditional and 0 for the other conditional over all four contents; for unknown content, it was 40 for both conditionals; for unrelated content it was 0 for both conditionals. A one-way repeated-measures ANOVA on the means of the degrees of truth participants assigned to the conditionals showed a significant difference between the three categories of content, F (2, 90) = 18.878, p < .0001, η_p_^2^=.296. The mean degrees of truth participants assigned to conditionals were higher for the *known* content compared to the *unknown* content, t (45) = 2.984, p < .005, CI [25.01342,128.8996], unlike the previous experiment; and higher for the *known* content compared to the *unrelated* content, t (45) = 7.013, p < .0001, CI [106.9204, 193.0796]; and for the *unknown* content compared to the *unrelated* content, t (45) = 2.831, p < .007, CI [21.08151, 125.0054], see Fig. 3a.

To explore the patterns of judgments further, and for comparison with the previous experiment, participants’ choices on the scale were transformed to a binary judgment by categorising responses of 100, 80 and 60 as judgments of truth, and responses of 0, 20 or 40 as judgments of falsity. Participants’ responses were scored for each of the 12 conditional pairs as, (a) true with one outcome only, i.e., 0, 20 or 40 for one conditional, and 100, 80 or 60 for the other, (b) true with either outcome, i.e., 60, 80 or 100 for each conditional, or (c) true with neither outcome, i.e., 0, 20 or 40 for each conditional. The one-way repeated-measures ANOVA on participants’ responses that only one outcome is true showed a significant difference between the three categories of content, F (2, 90) = 54.912, p < .0001, η_p_^2^ = .55, see Fig. 3b. Participants judged only one outcome as true more often for the *known* content compared to the *unknown* content, t (45) = 9.795, p < .0001, CI [1.70963, 2.59472], and the *unrelated* content, t (45) = 8.827, p < .0001, CI [1.3591, 2.16264]; and there was no difference between the *unrelated* and the *unknown* content, t (45) = 1.66, p = .104, CI [-0.86594, 0.08333].^2^

The results show that participants are willing to assess the degrees of truth of an impossible conditional, and their judgments of the degrees of truth of impossible conditionals are similar to their judgments of whether they could be true. The similarities are notable for their assessments for the *known* content that only one outcome is true, and for the *unrelated* content that neither outcome is true; however their assessments for the *unknown* content of the degrees of truth that both outcomes *are* true are reduced compared to their assessments that both outcomes *could be* true. Before considering the implications of the results, the next experiment tests whether participants are willing to make *definite* judgments that an impossible conditional *is* true or *is* false.

## Experiment 3

The experiment tested participants’ assessments of the truth of an impossible conditional when they make a definite judgment about whether it *is* true or *is* false. Once again, it tests that participants judge that a conditional is true with one outcome only, given *known* content; that it is false with either outcome, given unrelated content; and that it is true with either outcome, given *unknown* content.

### Method

#### Participants

The participants were a new set of 50 volunteers recruited from Prolific, who had not taken part in the previous experiment, including 30 women, 19 men, and one person who did not provide gender information, and they had an average age of 38 years, with a range from 18 to 75 years. No participants failed the attention checks so none were eliminated.

#### Materials, design and procedure

The materials were the same as the previous experiments, but the task was different. Participants made a definite judgment that the impossible conditional is true or false:

For the sentence below please decide whether it is true or false. Please read the sentence carefully.

If people were made of steel, they would bruise easily. 

The design and procedure of the experiment were the same as the previous experiments.

### Results and discussion

**Fig. 4:**
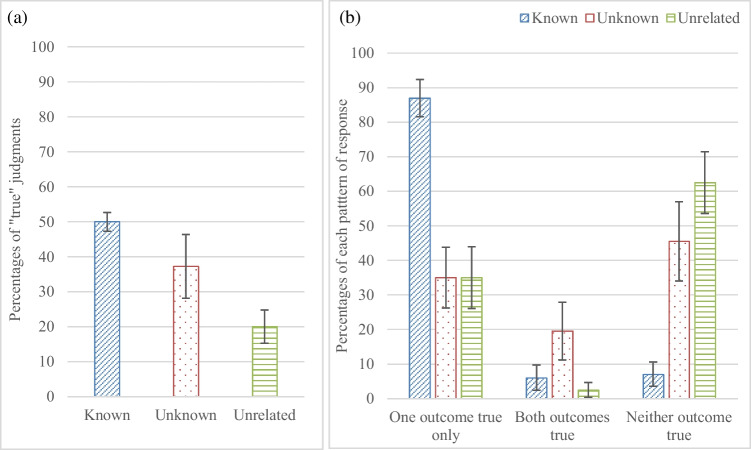
(**a**) The percentages of judgments that an impossible conditional is true for the three contents of Experiment 3 in which participants made judgments that an impossible conditional *is* true or *is* false, and (**b**) the percentages of responses scored for each pair of conditionals that they are true with one outcome only, with both outcomes, or with neither outcome, for the three contents. Error bars are 95% confidence intervals

A one-way repeated-measures ANOVA on the number of judgments participants made that a conditional is true showed a significant difference between the three categories of content, F (1.62, 79.369) = 26.902, p < .0001, η_p_^2^=.354. Participants judged conditionals true more often for the *known* content compared to the *unknown* content, t (49) = 2.872, p < .006, CI [0.30635, 1.73365], similar to the previous experiment, and more often for the *known* content compared to the *unrelated* content, t (49) = 10.112, p < .0001, CI [1.92306, 2.87694]; and for the *unknown* content compared to the *unrelated* content, t (49) = 3.673, p < .001, CI [0.62504, 2.13496], see Fig. 4a.

To explore the patterns of judgments further, participants responses were scored for each of the 12 conditional pairs that the conditionals are, (a) true with one outcome only, (b) true with either outcome, or (c) true with neither outcome. A one-way repeated-measures ANOVA on participants’ responses that only one outcome is true showed a significant difference between the three categories of content, F (2, 98) = 74.29, p < .0001, η_p_^2^ = .603, see Fig. 4b. Participants judged that only one outcome is true more often for the *known* content compared to the *unknown* content, t (49) = 9.742, p < .0001, CI [1.65095, 2.50905], and the *unrelated* content, t (49) = 11.835, p < .0001, CI [1.72681, 2.43319]; there was no difference between the *unknown* and *unrelated* content, t (49) = 0.^3^

The results show that participants are willing to make *definite* judgments that an impossible conditional *is* true or *is* false, and their judgments are similar to their judgments of whether an impossible conditional could be true or could be false, and their judgments of their degrees of truth. Once again, the similarities are notable for their assessments for the *known* content that only one outcome is true, and for the *unrelated* content that neither outcome is true; their assessments for the *unknown* content that both outcomes *are* true are reduced compared to their assessments that both outcomes *could be* true, similarly to their assessments of degrees of truth. The final experiment considers one other content for which participants may judge that both conditionals could be true.

## Experiment 4

The experiment tested assessments of the truth of an impossible conditional that contained a concept combination, such as *“If sheep and wolves were alike, they would eat a lot of grass”*. It tested judgments about whether the conditionals *could be* true or *could be* false. Once again, if participants attempt to use their real-world knowledge to construct a consistent simulation of an impossible conjecture and its suggested outcome, then they will judge that a conditional could be true with one outcome only, given *known* content, and it could be false with either outcome, given *unrelated* content; they will judge that it could be true with either outcome, given the new *concept combination* content.

### Method

#### Participants

The participants were a new set of 40 volunteers recruited from Prolific, who had not taken part in the previous experiment, including 24 women and 16 men, and they had an average age of 33 years, with a range from 18 to 57 years. No participants failed the attention checks so none were eliminated.

#### Materials, design and procedure

The task was the same as the first experiment, and the materials for the *known* and *unrelated* conditions were the same as the previous experiments. Instead of the materials for the *unknown* condition, materials for a new *concept combination* condition were included (see Table 1). The design and procedure of the experiment were the same as the previous experiments.

### Results and discussion

**Fig. 5:**
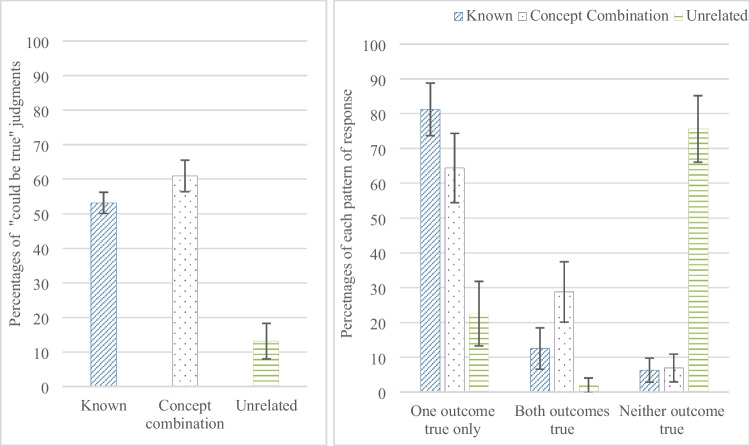
(**a**) The percentages of judgments that an impossible conditional could be true for the three contents of Experiment 4 including concept combination content, in which participants made judgments that an impossible conditional *could be* true or *could be* false, and (**b**) the percentages of responses scored for each pair of conditionals that they could be true with one outcome only, with both outcomes, or with neither outcome, for the three contents. Error bars are 95% confidence intervals

A one-way repeated-measures ANOVA on the number of judgments participants made that a conditional could be true showed a significant difference between the three categories of content, F (1.651, 64.391) = 165.892, p < .0001, η_p_^2^ = .81. Participants judged conditionals could be true more often for the *concept combination* content compared to the *known* content, t (39) = 3.665, p < .001, CI [-0.96997, -0.28003], similar to the first experiment; and more often for the *concept combination* content compared to the *unrelated* content, t (39) = 14.552, p < .0001, CI [3.26284, 3.29335]; and for the *known* content compared to the *unrelated* content, t (39) = 13.758, p < .0001, CI [2.72952, 3.67048], see Fig. 5a.

To explore the patterns of judgments further, participants responses were scored for each of the 12 conditional pairs that the conditionals could be, (a) true with one outcome only, (b) true with either outcome, or (c) true with neither outcome. A one-way repeated-measures ANOVA on participants’ responses that only one outcome could be true showed a significant difference between the three categories of content, F (1.507, 58.791) = 55.108, p < .0001, η_p_^2^ = .586, see Fig. 5b. Participants judged that only one outcome could be true more often for the *known* content compared to the *concept combination* content, t (39) = 4.076, p < .0001, CI [.34006, 1.00994], and the *unrelated* content, t (39) = 10.307, p < .0001, CI [1.8881, 2.81119]; and more for the *concept combination* content than the *unrelated* content, t (39) = 5.923, p < .0001, CI [1.10295, 2.24705].^4^

The results show that participants sometimes judged that an impossible conjecture about a combined concept could be true with either outcome, and their judgments are similar in this respect to their judgments of an *unknown* conditional. They also sometimes judged that an impossible conjecture about a combined concept could be true with just one outcome, whereas in the previous experiments they sometimes judged an *unknown* conditional could be false with both outcomes.

## General discussion

 I suggested as a starting hypothesis that people simulate impossible conjectures as if they are possible, relying on knowledge of reality to constrain their interpretation of them as true or false. The results of four experiments provide support for this proposal. Participants judged an impossible conjecture to be true with only one outcome when they could construct a consistent simulation for one of the conditionals, for example, “*if lakes were made of bleach, people would not swim in them*”, but not for the other, for example, “*if lakes were made of bleach, people would swim in them*”. Their knowledge about the real world guided their simulation of the outcome, for example, people swimming in a substance, consistent with a salient property of its conjecture, for example, the toxicity of bleach, and false with the other outcome. They judged an impossible conjecture false with both outcomes when they could not construct a consistent simulation for either one, for example, “*if trees were just like grass, their noses would be made of chickpeas”* and “*if trees were just like grass, their noses would not be made of chickpeas”,* given that the conjecture about similarity of trees and grass, and the outcome of noses made of chickpeas, is unrelated. But they tended to sometimes judge an impossible conjecture true with both outcomes when they could construct a consistent simulation for each one, for example, “*if Aristotle were identical to Heraclitus, he would be tall”* and “*if Aristotle were identical to Heraclitus, he would not be tall*”. They were able to construct a simulation of each outcome consistent with the conjecture, since the outcome is about a property unknown in reality (e.g., Heraclitus’s height). They also did so for concept combination content, such as “*if lobsters were the same as birds, they would make cosy nests”* and “*if lobsters were the same as birds, they would not make cosy nests”*. In such cases they were able to construct a simulation of each outcome consistent with the conjecture, since the concept combination in the conjecture could be interpreted to be about one concept or the other (e.g., about lobster-like birds, or bird-like lobsters).

The results were replicated for judgments of increasing strength, that the impossible conditional *could be* true (Experiments 1 and 4), has some degree of truth (Experiment 2), and *is* true (Experiment 3). However, judgments for an *unknown* outcome, for example, about Heraclitus’s height, and a *combined concept*, for example, lobster and birds, that both outcomes *could be* true were notably higher than judgments of the degrees of truth that both outcomes *are* true, or judgments that both outcomes *are* true. Although people appear willing to entertain the possibility that both outcomes could be true, they are unwilling to commit with any certainty to the conclusion that both outcomes *are* true. For impossibilities about *unknown* content*,* their simulations cannot be guided by knowledge of reality (they do not know Heraclitus’s height), and so their judgments of what *is* true are more conservative than their judgments of what *could be* true. Nonetheless, participants do not seem to consider that anything other than picking one answer as true is wrong, even when they are asked to state definitively in effect whether *X* or *not-X* is true.

Moreover the observation that people assess that impossible conjectures with *unknown* content, or *concept combination* content, could be true for both outcomes, indicates they are not at the mercy of a cognitive bias that prevents them from recognising impossible conjectures could be true with both outcomes. It undermines the proposal that such a cognitive bias underlies their judgments about impossible conjectures with *known* content, that they are true for only one outcome (cf. Williamson, 2018). It may even cast doubt on the idea that they rely on such a conditional probability calculation, if such a view were to commit them to identify only one of a pair of conditionals with opposite outcomes as true.

As a brief aside, given the current interest in the Artificial Intelligence reasoning capabilities of Large Language Models, it is notable that given the tasks in the four experiments reported here, the responses of ChatGPT appear to differ from those provided by human participants, and from those prescribed by prominent logical analyses. It responded to several instances of *known* content conditionals by indicating one could be true and one could be false; to instances of *unknown* content and instances of *concept combination* content by indicating one could be true and one could be false, or both could be false; and to instances of *unrelated* content by indicating both could be false. It did not provide the response that both conditionals could be true to any instances.

Conditionals with impossible conjectures differ from counterfactuals. People often create counterfactuals to explain events, for example, “*if I had read more textbooks, I would have passed the exam*” (e.g., Mandel & Lehman, 1996; Walsh & Byrne, 2007; see also Kirfel et al., 2022), sometimes by identifying causal links between the conjecture and its outcome (e.g., Frosch & Byrne, 2012; Lucas & Kemp, 2015; Lagnado et al., 2013; McEleney & Byrne, 2006; Meder, et al., 2009). The counterfactual can help them learn to prevent an outcome by providing a blueprint for future intentions, for example, *“I will read more textbooks next time”* (Ferrante et al., 2013; Roese & Epstude, 2017). But people do not tend to explain outcomes with “miracle world” impossible conjectures, for example, *“if there hadn’t been gravity, the 'plane wouldn’t have crashed*” (Lebow, 2000). Why then do people ever make assertions about impossible conjectures? Of course, they sometimes entertain an impossible conjecture without intending it literally, for example, “*if I were you…”,* instead communicating it as short-hand for a perspectival shift, “*if it were me in your situation…*”. But they rely on other impossible conjectures, for example, “*if pigs could fly…”,* or “*if hell were to freeze over…*” to illustrate the implausibility of an outcome. A key function of such an impossible conditional may be to rule out its hypothetical outcome, to emphasise it as untrue. The results here imply that people can find them useful, not only in the domain of science (e.g., Jenny, 2018; McLoone, 2020; McLoone et al., 2023; Tan, 2019), but also in everyday life.

## Footnotes

1 A consistent pattern holds regardless of whether we analyse judgments that only one outcome could be true, both could be true, or neither could be true. An ANOVA instead (since the responses are not independent) on responses that both outcomes could be true also showed a significant difference, F (1.584, 76.055) = 54.311, p < .0001, η_p_^2^ = .531. Participants judged both outcomes could be true more often for the *unknown* content compared to the *known* content, t (48) = 6.435, p < .0001, CI [-2.14276, -1.12255], and the *unrelated* content, t (48) = 9.471, p < .0001, CI [1.70404, 2.62249]; and more often for the *known* than the *unrelated* content, t (48) = 3.433, p < .001, CI [0.2198, 0.84142]. Similarly, an ANOVA on responses that neither outcome could be true also showed a significant difference, F (1.682, 80.752) = 92.36, p < .0001, η_p_^2^ = .658, see Fig. 2. Participants judged neither outcome could be true more often for the *unrelated* content compared to the *known* content, t (48) = 11.889, p < .0001, CI [-2.55297, -1.81437], and the *unknown* content, t (48) = 9.65, p < .0001, CI [-2.39206, -1.56713]; there was no difference between the *known* and *unknown* content, t (48) = 1.493, p = .142. For three such non-independent ANOVAs the Bonferroni corrected alpha is p < .0056.

2 An ANOVA instead (since the responses are not independent) on participants’ responses that both outcomes are true showed a significant difference, F (1.306, 58.786) = 6.215, p < .01, η_p_^2^ = .121, see Fig. 3. Participants judged both outcomes are true more often for the *unknown* content compared to the *known* content, t (45) = 2.782, p < .008, CI [-0.67461, -0.10800], and the *unrelated* content, t (45) = 2.46, p < .018, CI [0.06699, 0.67214]; there was no difference between the *unrelated* than the *known* content, t (45) = 0.33, p = .743. Similarly, an ANOVA on participants’ responses that neither outcome is true showed a significant difference, F (2, 90) = 36.207, p < .0001, η_p_^2^ = .446, see Fig. 3. Participants judged neither outcome is true more often for the *unrelated* content compared to the *known* content, t (45) = 6.762, p < .0001, CI [-2.28535, -1.23638], and for the *unknown* content compared to the *known* content, t (45) = 8.589, p < .0001, CI [-2.14695, -1.33131]; there was no difference between the unrelated and the *unknown* content, t (45) = .088, p = .93. For three such non-independent ANOVAs the Bonferroni corrected alpha is p < .0056.

3 An ANOVA instead (since the responses are not independent) on responses that both outcomes are true also showed a significant difference, F (1.311, 64.217) = 14.216, p < .0001, η_p_^2^ = .225, see Fig. 4. Participants judged both outcomes are true more often for the *unknown* content compared to the *known* content, t (49) = 3.764, p < .0001, CI [-.82829, -.25171], and the *unrelated* content, t (49) = 4.056, p < .0001, CI [.34305, 1.01695]; there was no difference between the *known* and the *unrelated* content, t (49) = 1.851, p = .07. Similarly, an ANOVA on responses that neither outcome is true also showed a significant difference, F (2, 96) = 48.04, p < .0001, η_p_^2^ = .495, see Fig. 4. Participants judged neither outcome is true more often for the *unrelated* content compared to the *known* content, t (49) = 11.675, p < .0001, CI [-2.60211, -1.83789], and the *unknown* content, t (49) = 2.74, p < .0009, CI [-1.1787, -.1813]; and more often for the *unknown* content than the *known* content, t (49) = 6.095, p < .0001, CI [-2.04777, -1.03223]. For three such non-independent ANOVAs the Bonferroni corrected alpha is p < .0056.

4 An ANOVA instead (since the responses are not independent) on responses that both outcomes could be true also showed a significant difference, F (1.592, 62.104) = 32.33, p < .0001, η_p_^2^ = .451, see Fig. 5. Participants judged both outcomes could be true more often for the *concept combination* content compared to the *known* content, t (39) = 4.759, p < .0001, CI [-.92626, -.37374], and the *unrelated* content, t (39) = 6.649, p < .0001, CI [.74799, 1.40201]; and more for the *known* than the *unrelated* content, t (39) = 4.226, p < .0001, CI [.2216, .6284]. Similarly, an ANOVA on responses that neither outcome could be true also showed a significant difference, F (1.316, 51.331) = 151.232, p < .0001, η_p_^2^ = .795, see Fig. 5. Participants judged neither outcome could be true more often for the *unrelated* content compared to the *known* content, t (39) = 13.393, p < .0001, CI [-3.19411, -2.35589], and the *concept combination* content, t (39) = 12.5, p < .0001, CI [-3.19498, -2.30502]; there was no difference between the *known* content and the *combined concept* content, t (39) = 0.255, p = .80. For three such non-independent ANOVAs the Bonferroni corrected alpha is p < .0056.
